# On the importance of local dynamics in statokinesigram: A multivariate approach for postural control evaluation in elderly

**DOI:** 10.1371/journal.pone.0192868

**Published:** 2018-02-23

**Authors:** Ioannis Bargiotas, Julien Audiffren, Nicolas Vayatis, Pierre-Paul Vidal, Stephane Buffat, Alain P. Yelnik, Damien Ricard

**Affiliations:** 1 CMLA, ENS Cachan, CNRS, Université Paris-Saclay, 94235 Cachan, France; 2 COGNAG-G UMR 8257, CNRS, SSA, Université Paris Descartes, Paris, France; 3 Institut de Recherche Biomédicale des Armées Brétigny, Bretigny-sur-Orge, France; 4 École du Val-de-Grâce, Service de Santé des Armées, Paris, France; 5 PRM Department, GH Lariboisière F. Widal, AP-HP, Paris Diderot University, UMR 8257, Paris, France; 6 Service de Neurologie, HIA Percy, Service de Santé des Armées, Clamart, France; University of Illinois at Urbana-Champaign, UNITED STATES

## Abstract

The fact that almost one third of population >65 years-old has at least one fall per year, makes the risk-of-fall assessment through easy-to-use measurements an important issue in current clinical practice. A common way to evaluate posture is through the recording of the center-of-pressure (CoP) displacement (statokinesigram) with force platforms. Most of the previous studies, assuming homogeneous statokinesigrams in quiet standing, used global parameters in order to characterize the statokinesigrams. However the latter analysis provides little information about local characteristics of statokinesigrams. In this study, we propose a multidimensional scoring approach which locally characterizes statokinesigrams on small time-periods, or *blocks*, while highlighting those which are more indicative to the general individual’s class (faller/non-faller). Moreover, this information can be used to provide a global score in order to evaluate the postural control and classify fallers/non-fallers. We evaluate our approach using the statokinesigram of 126 community-dwelling elderly (78.5 ± 7.7 years). Participants were recorded with eyes open and eyes closed (25 seconds each acquisition) and information about previous falls was collected. The performance of our findings are assessed using the receiver operating characteristics (ROC) analysis and the area under the curve (AUC). The results show that global scores provided by splitting statokinesigrams in smaller blocks and analyzing them locally, classify fallers/non-fallers more effectively (AUC = 0.77 ± 0.09 instead of AUC = 0.63 ± 0.12 for global analysis when splitting is not used). These promising results indicate that such methodology might provide supplementary information about the risk of fall of an individual and be of major usefulness in assessment of balance-related diseases such as Parkinson’s disease.

## Introduction

Postural control is defined as the ability of individuals to maintain a controlled upright position. It is achieved by combination of visual, proprioceptive, and vestibular system [1]. Impairments and disorders such as myoskeletal disorders, visual, balance or gait impairments associated with aging may progressively worsen the individual’s postural control, increasing consequently the risk of falling [2]. Falls are considered as one of the major causes of injury in elderly, resulting in further mobility restriction, autonomy problems in daily activities (bathing, cooking, etc) or even death [3, 4]. Therefore, prevention of falling through prediction and accurate evaluation of risk has become an important issue considering that the one third of population > 65 years old faces at least one fall per year [4].

A tool of choice for clinical researchers to quantify and evaluate postural control are force platforms. Such platforms record the displacement of the centre of pressure (CoP) which is applied by the whole body in time [5] while the individual stands quietly upon it and follows the clinician’s instructions/protocol. This measurement is usually called statokinesigram. Statokinesigrams have been previously used in assessing balance disorder in healthy or balance-related disease populations [6, 7]. Since CoP platforms became more portable and accessible, there has been an increasing interest in exploiting all available information of the statokinesigrams. Many indices derived by the CoP displacement have been proposed previously, showing that CoP displacement characteristics and dynamic structure(ex.regularity) can reflect individuals’ postural impairement [3, 8–12]. Recently, it has been shown through frequency analysis that energy distribution at specific frequency-bands, can inform about the chosen postural strategy by an individual [13]. Also, researchers analyzed statokinesigrams using time-scale wavelet analysis [14–16], in order to evaluate as well as detect the transitions between postural strategies.

The variety of methods indicates that clinical research becomes more and more interested about the evaluation of postural control. However, there is no agreement neither in healthy nor in non-healthy populations [17–19] whether these features or transformations alone are able to fully assess the individual’s posture control.

Therefore, recent works [20–22] proposed a combination of multiple global features derived from the statokinesigrams in order to classify fallers and non-fallers. In one of our previous studies [22] we observed that although none of the indices alone could classify effectively fallers/non fallers (weak classifiers), the combination of all features through non-linear multi-dimensional classification showed significant results. This result indicates that the postural control is better characterized by a combination of statokinesigram elements (a “profile”) rather than one dominant index (either structural or dynamic). We believe that the above multi-dimensional approaches open new perspectives in the analysis of complex phenomena such as postural control.

Additionally, statokinesigrams of an individual may not necessarily have a uniform faller/non-faller profile, since the statokinesigram dynamics change throughout its duration—for instance, a faller may maintain a “non-faller profile” for a significant part of his/her statokinesigram.

The aforementioned multi-dimensional approaches characterized globally the statokinesigrams, an approach which relies on the implicit assumption that the signal may present an homegeneous profile, but provided little local information about particular parts of the signals—or *blocks*. Therefore, although the latter methods presented major usefulness, they did not provide insights about which parts are more indicative of the subject’s class (faller/non-faller).

In this work, we assumed that statokinesigrams are heterogeneous and that blocks with significantly different characteristics (quiet and unquiet blocks—QBs and UBs correspondingly from now on) are expected to co-exist in most of the cases. We hypothesized that in quiet-stand, UBs will be more prominent and frequent in fallers’ statokinesigrams than in those of non-fallers and consequently this fact will affect significantly individuals’ global score of postural control. As far as we know, such hypothesis has never been formally proposed or evaluated using mathematical models. Therefore, the objective of this work is to highlight the existence of UBs, both in faller and non-faller statokinesigrams, as well as to quantify their influence on the individual’s profile. In order to evaluate the latter hypothesis, our approach followed the steps below:

1. The separation of statokinesigrams into blocks of predefined time-periods,
2. The description of every block with a simple three-dimensional description, using three well-known and established indices from the literature,
3. The clustering of the derived blocks, into two clusters (QBs/UBs)
4. The evaluation of the relevance of those through a global score per individual using a naive classification approach.

## Methods

We included 126 subjects (78.5 ± 7.7 years, 80 females) from the Neurology department of the HIA Percy hospital (Clamart, France) and the consultation office of a practitioner (Paris, France). Inclusion criteria: Participants (1) had age > 65 years, (2) were addressed in routine consultation in general medicine or neurology, (3) did not present any clinical sign of balance impairment (vestibular, cerebellar, proprioceptive) even if they had fallen during the last 6 months (4) were able to stand on the platform, (5) gave informed consent. Particularly, only asymptomatic individuals after clinical examination were considered to this study. Individuals which were significantly hypertensive (mean Systolic Blood Pressure (SBP) ≥ 140 mmHg or mean Diastolic Blood Pressure (DBP) ≥ 90 mmHg), hypotensive (SBP ≤ 90 mmHg or DBP ≤ 60 mmHg), had particular impairements or used medication which could alter significantly their balance (such as vasoactive, phychotrope drugs) were excluded. Moreover, characteristics such as weight, height and principal syndromes were collected (Table 1). The clinical trial registered at ANSM (ID RCB 2014-A00222-45) was approved by the following ethics committee/institutional review board(s): (1) Comité de protection des Personnes (CPP), Ile de France Paris VI, (2) Agence National de Sécurité du médicaments et des produits de santé (ANSM), (3) Commission Nationale de l’Informatique et des Libertés (study complies with the MR-001). After information and allowing adequate time for consideration, written informed consent was obtained before participants are included in the study.

**Table 1:** Demographic characteristics of the participants. Fallers are patient who declared at least one fall in the six previous months. No statistically significant difference was found between the two population regarding age, weight, height and body mass index (BMI).

	Total Sample	Non Fallers	Fallers
Demographic	126	108	18
Male	46	38	8
Female	80	70	10
Age (years)	78.5(±7.7)	77.2(±6.4)	79.2(±7.2)
Weight (kg)	69.6(±10.7)	69.4(±10.3)	70.0(±11.4)
Height (cm)	167.0(±8.0)	167.0(±8.0)	167.7(±8.3)
BMI (kg.*m*^−2^)	24.96(±2.4)	24.92(±2.3)	25(±2.3)

### Balance measurements

Balance measurements were acquired using a Wii Balance Board (WBB)(Nintendo, Kyoto, Japan) which has been found a suitable tool for the clinical setting with an acceptable accuracy [23–25]. The statokinesigrams were registered, recorded and saved in a distant database using a custom tablet application, in Android, specially developed for the study. The participants were asked to remove their shoes and step on the platform placing their feet in the most comfortable position without exceeding the shoulder width, and to stand in upright position with open eyes and the arms laying at the side. The trajectory of the CoP positions was recorded for 25 seconds. Subsequently, participants were asked to close their eyes. After a ten-second pause, clinical experts recorded 25 additional seconds with eyes closed. From each statokinesigram, the first and the last 2.5 seconds were excluded from further analysis (20 seconds per statokinesigram).

### Fall assessment

In order to classify the participants, a fall questionnaire was filled for each subject keeping information about previous falls during the last six months [26]. Following previous works, participants were classified as fallers if they came to a lower level on the ground unintentionally (at least one time during the last six months) [27]. Supplementary information about the falls (environment, circumstances, consequences) were registered. It is worth noting that in our population, fall-prone individual reported no more than two falls in the past six months.

### Preprocessing of trajectories

Since the WBB acquired the CoP trajectories using a variable time resolution, the obtained statokinesigrams were resampled at 25Hz using the SWARII algorithm previously proposed in [28]. Both open and closed-eyes were split in fixed-length time blocks, and on each time-block, the three following widely used indices [3, 22, 29] were calculated:

- The surface of the 95% confidence ellipse (**s**_95_)
- Mean of the velocity magnitude of the CoP displacement (**v**_*mean*_)
- Standard deviation of the medio-lateral (ML) displacement values (*σ*_*X*_)

These indices were then normalized across the current set of trajectories.

### Gaussian Mixture Model (GMM) algorithm

In order to cluster the resulting three dimensional description of the blocks, we used two Gaussian Mixture Model (GMM), one for the closed eyes trajectories, and one for the open eyes. In this section, we briefly discuss the ideas of GMM and we refer the reader to [30] for more details.

The Gaussian mixture models (GMM) is a classical unsupervised clustering algorithm. Given a number of cluster *k* (in our case, *k* = 2 for QB/UB), and a three dimensional set of points

$$\begin{array}{c} Z=(\begin{array}{c} s_{95}\\ v_{mean}\\ \sigma_{X} \end{array}) \end{array}$$

(here each point *Z*_*j*_ is the normalized indices of each block), it tries to construct a mixture of *k* multivariate Gaussian random variable, following the maximum likelihood principle. To achieve this, it proceeds with the expectation—maximization (EM) principle. First, the weights *π*_*i*_, centers ${\left(\mu_{i}\right)}_{i=1}^{k}$ and the covariance matrix ${\left(\Sigma_{i}\right)}_{i=1}^{k}$ of each Gaussian random variable $N_{i}$ are randomly initialized using the classical repetitive initialization procedure (20 times). Then the algorithm repeat the following steps until convergence:

- The probability *p*_*i*,*j*_ that the point *Z*_*j*_ belongs to the cluster *i*:
  $$\begin{array}{c} p_{i,j}=\frac{\frac{\pi_{i}}{\sqrt{|\Sigma_{i}|}}\exp(-{\left(Z_{j}-\mu_{i}\right)}^{T}\Sigma_{i}^{-1}\left(Z_{j}-\mu_{i}\right))}{\sum_{i}\frac{\pi_{i}}{\sqrt{|\Sigma_{i}|}}\exp(-{\left(Z_{j}-\mu_{i}\right)}^{T}\Sigma_{i}^{-1}\left(Z_{j}-\mu_{i}\right))} \end{array}$$
- The weights, centers and covariance matrices of the $N_{i}$ are updated according to the *p*_*i*,*j*_:
  $$\begin{array}{c} \pi_{i}=\frac{1}{n}\sum_{j}p_{i,j} \end{array}$$
  $$\begin{array}{c} \mu_{i}=\frac{1}{\sum_{j}p_{i,j}}\sum_{j}p_{i,j}Z_{j} \end{array}$$
  $$\begin{array}{c} \Sigma_{i}=\frac{1}{\sum_{j}p_{i,j}}\sum_{j}p_{i,j}{\left(Z_{j}-\mu_{i}\right)}^{T}\left(Z_{j}-\mu_{i}\right) \end{array}$$

The cluster maximizing the likelihood of the mean of the fallers profiles was labeled as quiet blocks (QBs), and the other as unquiet blocks (UB). This allows the computation of a score from the resulting clusters.

### Evaluation

**Fig 1:**
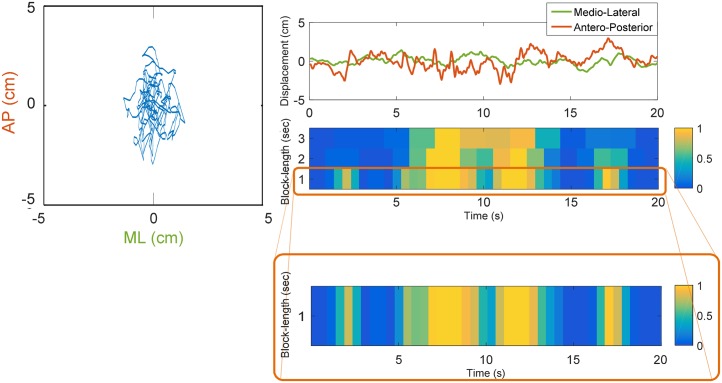
Representation of the score of the different blocks for a statokinesigram deduced from the CoP antero-posterior and medio-lateral using multiple block-lengths and 50% overlap. A large value (yellow colour) indicates a high probability of belonging to the UBs cluster, while a small value (blue colour) represents a block with low probability of belonging to UBs cluster—and therefore a large probability to belong to the QB cluster. Detail of the analysis using 1-second block-length is provided (orange box).

To evaluate the relevance of the local analysis, we proceeded as follows:

1. We used a classical machine learning approach on a train-test split. Available population was randomly split into a train-set, containing 70% of the statokinesigrams(without replacement), and a test-set containing the remaining 30%, keeping the same proportion of faller and non faller in every set.
2. Trajectories were separated using predefined block-length and overlap parameter (50%) and each block was pre-processed to obtain the three indices which were normalized before processed.
3. The normalized indices of the train set were used to train the GMM clustering algorithm, with two clusters (QBs/UBs).
4. The normalized indices of the test set were evaluated using the previously trained GMM. The score of each block was defined the probability of belonging to the UB cluster.
5. The score of a trajectory was computed as the average of the blocks’ scores, and the global score of an individual as the average of its open-eyes score and its closed-eyes score. Therefore, every block length Fig 1) provided an independent global score.

**Fig 2:**
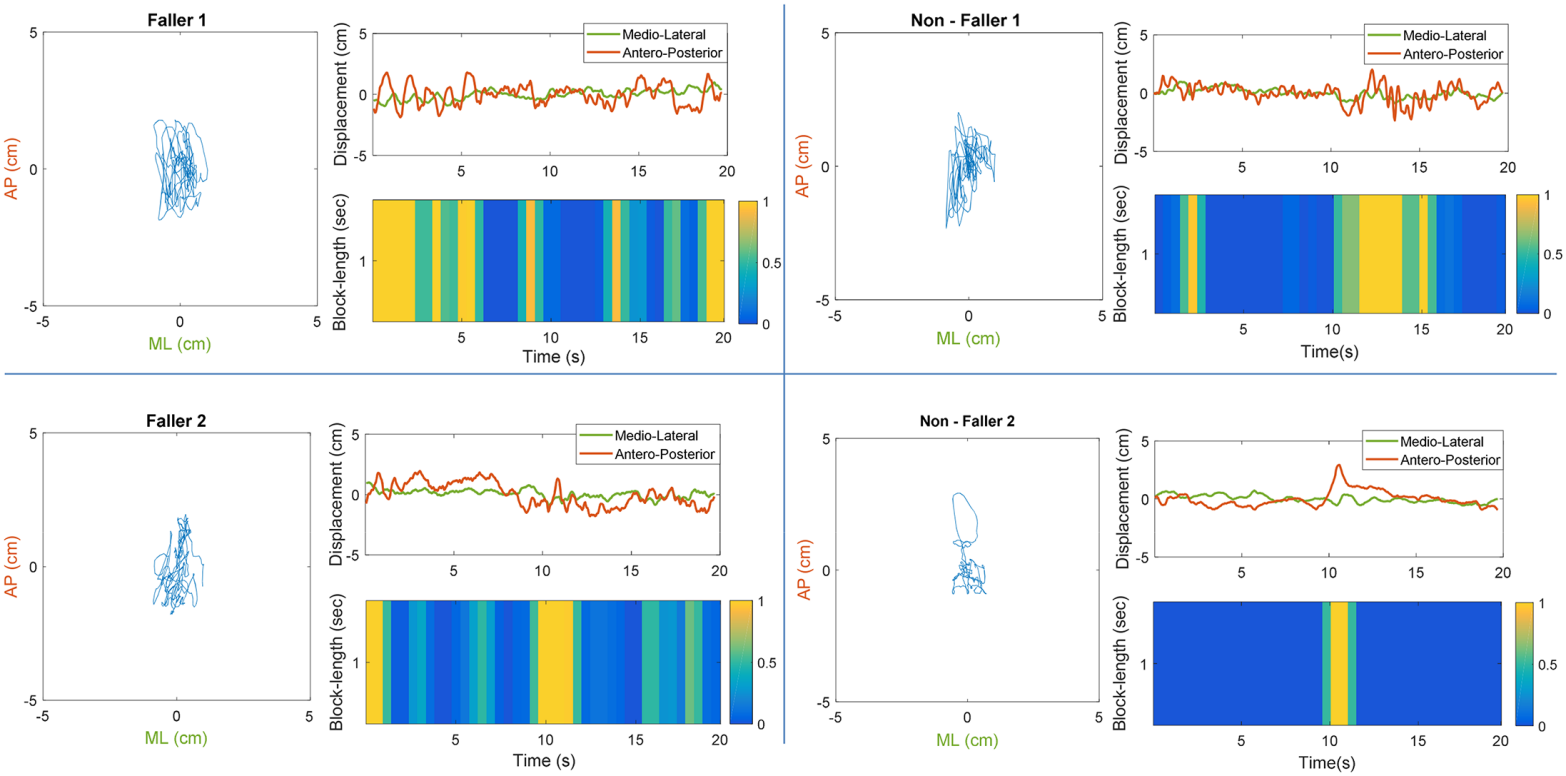
ROC curves obtained by the method for different block lengths. These curves are the average curves from the aformentioned 30 repetitions of the algorithm.

The prediction performance of global score of test-set was measured by receiver operating characteristics (ROC) analysis and its area under the curve (AUC). The performance was validated using the Monte Carlo Cross Validation (MCCV) approach [31] keeping the same proportion of faller and non faller in train/test set. The whole algorithm, proportional data-set splitting, block scoring, global scoring etc was run 30 times. For each data split, the classifier was retrained from scratch with the training examples and estimated its performance with the test examples. The true performance estimate was obtained as the average of the 30 separate performance estimates. The average AUCs and ROC curve were reported in (Table 2, Fig 2). Comparison between performances was performed using the Mann Whitney nonparametric test. The differences in performance were checked using the non-parametric Wilcoxon rank-sum test.

**Table 2:** Classification performance of the global score using different block-lengths. While reasonable lengths performed almost equally, when block separation is not used (processing raw signals as one block), area under the curve (AUC) was significantly lower. * indicates that AUC derived by no block-separation is significantly lower than the others (p<0.001).

Block—length	AUC (average ± std)
1 sec	0.77(±0.09)
2 sec	0.77(±0.10)
3 sec	0.76(±0.09)
Signal’s duration	0.63(±0.12)*

## Results

Individuals’ description including basic characteristics and body mass index (BMI) are summarized in Table 1.

**Fig 3:**
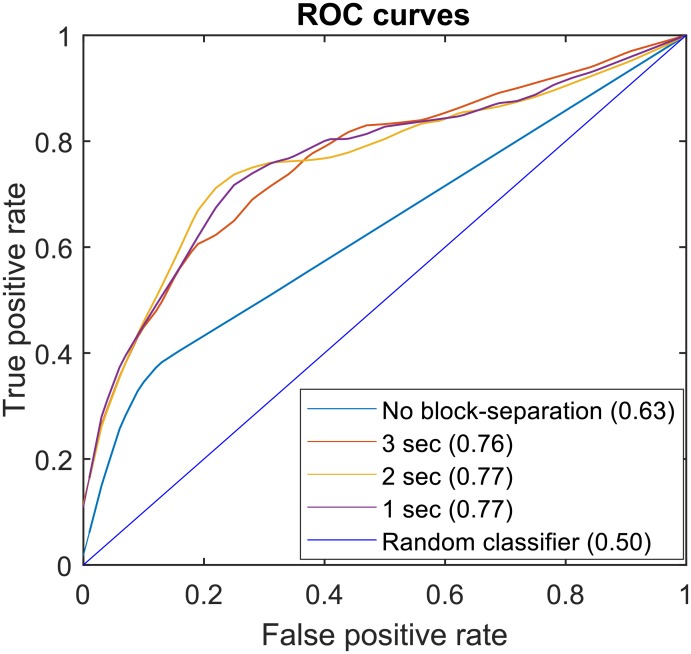
Representation of the analysis of four statokinesigrams that have not easily distinguishable statokinesigrams(two fallers, two non-fallers) deduced from the CoP antero-posterior and medio-lateral using block-length of 1 second. It can be observed that although all the examples include parts that are strongly classified as possible UBs, these parts are more frequent in fallers than in non-fallers.

Fig 1 shows a representation of the statokinesigrams derived from the score attributed to each time-block, for different choices of block-length and 50% overlap. This score indicates the probability of each time-block to belong to the UB cluster. The average performances of global scores over 30 train test split, are presented in Table 2. It can be noticed that the separation of statokinesigrams in time-blocks provide identical performances in all block-length choices. Fig 3 presents additional examples of the statokinesigram representation, using the 1-second-length blocks, for different fallers and non-fallers individuals.

The evaluation of each score in the classifying fallers and non-fallers among the test population gave the significant 0.76 − 0.77 average AUC (see Table 2 and Fig 2). This performance is robust to the choice of block-length.

However, when there is no block-separation (i.e. raw signals are processed as one block of length 20 sec), there is a decrease of classification performance (AUC = 0.63). In order to check for gender effect, males and females were also analyzed separately and found with almost identical performances.

Table 3 shows that when block-separation is used, both fallers/non-fallers present UBs. However this percentage is significantly higher in fallers than in non-fallers, explaining the above difference in final score between the two categories.

**Table 3:** Percentages of blocks per subject in test set classified as UBs with posterior probability >0.5 from acquisition with closed eyes. Block decomposition of statokinesigram showed that although both categories may have UBs, this percentage is significantly higher in fallers than in non-fallers, explaining the difference in final score between the two categories. * indicates statistical significance between fallers and non-fallers (p<0.001).

Block—length	Fallers (average ± std)	Non—fallers (average ± std)
1 sec	0.37 ± 0.16*	0.10 ± 0.04
2 sec	0.37 ± 0.16*	0.09 ± 0.04
3 sec	0.35 ± 0.17*	0.10 ± 0.04

**Fig 4:**
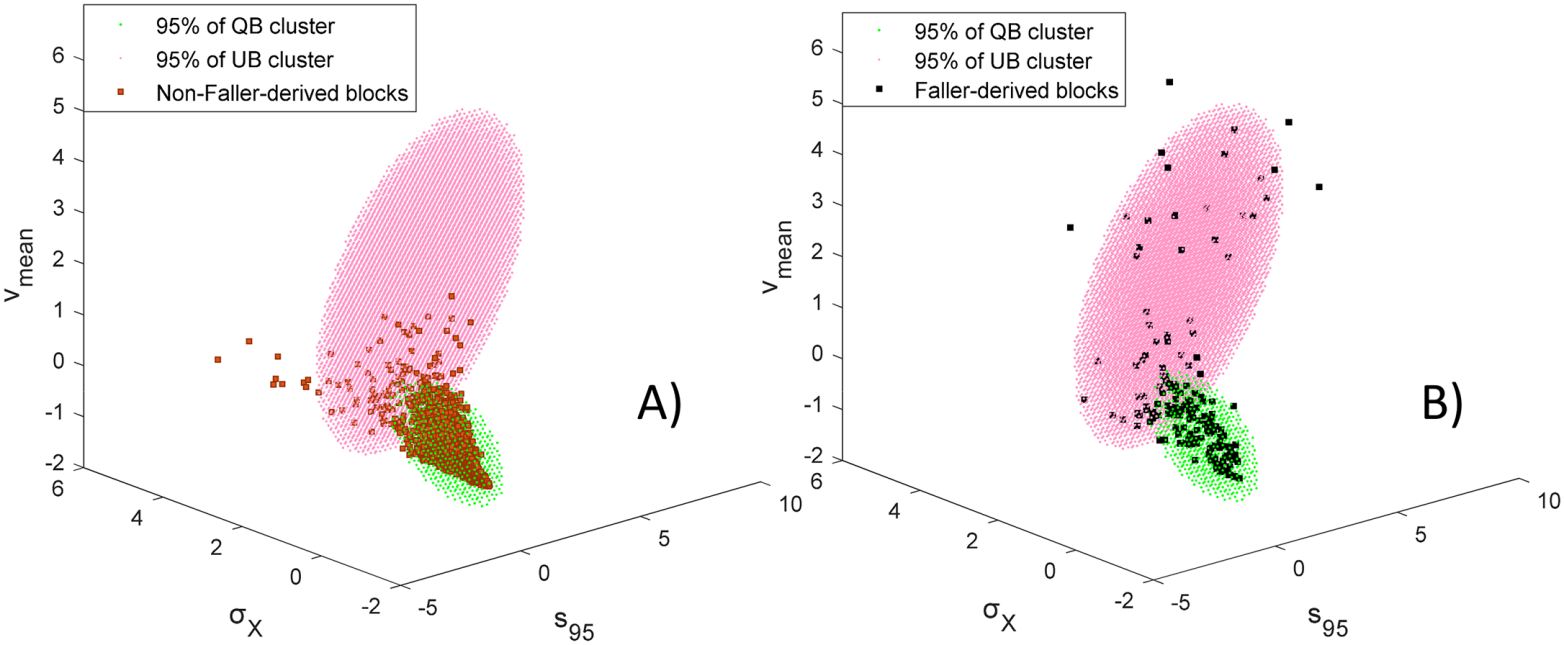
Probability density for the two-component mixture distribution (95%) and three-second-blocks of A) Non-fallers and B)Fallers, illustrated in the normalized 3-dimensional space. The two normal components overlap, but their centers are distinct. Many faller-derived periods are closer to the QBs component and conversely, many non-faller derived blocks are closer to the UBs centre.

Finally, Fig 4 illustrates the distribution of three dimensional representation for faller-derived and non-faller-derived blocks, as well as the the clusters derived by the GMM algorithm.

## Discussion

The objective of this study was to verify the hypothesis that 1) a statokinesigram is not necessarily uniform, in the sense that its dynamics may change throughout its duration, 2) quiet and unquiet blocks (called QBs and UBs in this work) are both present in most statokinesigrams, and 3) the presence and profile of UBs can be used to efficiently classify fallers and non fallers.

We introduced a new method for highlighting the parts of statokinesigram that are more prone to be UBs. This method combines: 1) the separation of statokinesigram in multiple time-blocks, 2) the computation of their multi-dimensional profile, using common indices from the literature, and 3) the model-based clustering of these profile in order to characterize every time-block of the statokinesigrams. This decomposition of the signal in blocks was aimed at the analysis of the local behavior of the CoP, and allows to study the dynamic changes throughout the statokinesigram. It resulted that 1) UBs exist in both fallers’/non-fallers’ statokinesigrams and 2) they represent a significantly higher percentage of the signal in fallers than in non-fallers. The global score (which is the average of block scores) was used in order to evaluate the effectiveness of the algorithm through global classification of population (fallers/non-fallers). Various block lengths (1, 2 and 3 seconds) resulted in a global score with nearly identical classification performance (0.76 <AUC< 0.77) while the resulted global score without block decomposition showed significantly lower performance (AUC = 0.63).

### Statokinesigram and indices

The CoP acquisition platforms have been widely used in biomedical and clinical studies in order to further assess the postural control of an individual [5, 7]. The choice of the features used in our work was based on previous clinical works which reported that statokinesigrams medio-lateral and antero-posterior variation, trajectories’ velocity, acceleration or CoP’s trajectory area [3, 9, 32], might classify CoP trajectories by posture quality. As mentioned in the introduction, there is no agreement about the best indices that must be used. Previous works [6, 33] raised some concerns about the ability of single index alone, despite of their general usefulness (e.g. medio-lateral movement), to evaluate the risk of falling or to distinguish effectively a faller by a non faller. Recently, some works used multi-dimesnional analysis in order to characterize postural control [29, 34] and, combined with machine learning approaches, showed significant improvement of the classification’s performance [20–22]. The idea behind these approaches is that multiple characteristics affect participant’s statokinesigram simultaneously interacting with each other. It has been shown clearly [22] that using combinations of -even- weak classifiers can distinguish effectively fallers’ by non-fallers’ statokinesigrams. Therefore, a reasonable number of characteristics needs to be analyzed as an ensemble instead of one by one separately.

### Methodological choices

In line with the above, the proposed method is inspired by the advantages of the multi-dimensional analysis and machine learning techniques, integrating the idea of a time-scale analysis of a signal (like wavelet analysis used previously in statokinesigrams [14, 16]).

#### EM for Gaussian mixture model

Our initial assumption was that the statokinesigrams are heterogeneous, i.e. that a single signal contains both “quiet” (QBs) and “unquiet” (UBs) blocks. This assumption leads to the natural choice of a clustering approach where the labels of each block are not known in advance. (See S1 Appendix for a comparison of algorithms when statokinesigrams are considered homogeneous) To study the presence of different types of dynamics, we used a Gaussian Mixture Model (GMM), a standard clustering algorithm. Due to the strong non convexity of the optimization problem minimized by the GMM, we proceeded to multiple repetition (20) of the algorithm in order to increase the chance to find a nearly optimal solution (with respect to the maximum likelihood criterion). It is a classical approach for this algorithm in multivariate context [35].

The result of the GMM algorithm were consistent over multiple trials, and highlighted the existence of two distinctive clusters (see Fig 4). The two clusters were identified *a posteriori* as quiet and unquiet blocks respectively, due to the fact 1) the non-faller-derived blocks were more represented in the first cluster (green), and 2) the blocks in the second cluster (red) were generally characterized by higher speed, larger standard deviation of ML movement and larger surface coverage. Therefore, the blocks belonging to the second cluster (red) were referred as UBs.

#### Existence of “UBs”

The wide term “unquiet blocks” (UBs) might involve periods of transition between strategies, exploration periods, shifting of weight to one foot, head movement or even instability periods that might occur even if subjects are in standing position without disturbance. However, it could be expected that-even-those periods would be minor in individuals that are able to follow the acquisition protocol and have relatively good postural control.

Although the latter assumption remains without hard clinical evidences or annotations, it seems that it is generally confirmed by our results. Indeed, in the non-faller case, these parts were most of the times rare. Conversely, fallers presented a significantly higher percentages (≈ 0.35%) of UBs (see Table 3). Moreover, since our approach resulted a relatively high performance when block-separation is applied (see Table 2), it seems that our hypothesis about the significant presence of UBs in subjects with low postural control is confirmed. Future efforts should be focused on the finer definition of the “UBs”. Observational annotations by an expert which would take into account the individual’s postural behavior, locally (in time) as well as globally, could also help in creating statistical profiles of possible UBs or QBs.

#### Choice of block-length

Although UBs and balance recoveries appears to exist in both fallers and non-fallers’ statokinesigrams, their duration vary. So the choice of the ideal block length is not a trivial task. We proposed a multi-scale analysis, giving the opportunity to the clinicians to investigate particular phenomena if they have prior knowledge about their approximate duration.

It is known that age affects drastically (decreases) the sensory response to a small “unstable” moment that may be presented in quiet stand of the elderly. So, a relative slowness in recovery [36] is expected, possibly as “strategy of choice” in order to avoid disequilibrium (uncontrolled passings of the stability borders) [37]. Since the average age of our population was relatively high (78.5 ± 7.7), we expected to find slightly better classification performances in larger block lengths. However, global scores resulted equal classification performance in all block-lengths (Table 2). A possible reason for this result might be that quiet standing might be insufficient exercise in order to have a full expression of the aforementioned slowness in recovery or strategy-alteration of elderly individuals.

Technically, there is no guarantee that all populations will present equal prediction performance for every block-length choice. For example richer acquisition protocols, particular examined diseases or syndromes may present specific types or duration of investigated periods [36]. Further investigation in various populations is needed in order to clarify the effect of block-length to the classification performance. Detailed clinical/observational annotation during the statokinesigrams’ recordings by an expert could also significantly contribute to the ideal choice of block-lengths.

Remarkably, Table 2 showed that when block-separation is not applied, classification performance drops significantly. This might indicate that considering only global indices of statokinesigrams, a significant amount of information regarding the local aspects, that might be indicative of the subject’s profile, is lost. Indeed, the influence of the presence of unusual values of the indices for short periods of time, are often “smoothed” or “averaged” when considered over the entire signal, resulting in a minor contribution to the final global index. Therefore, while different block lengths might give satisfying results, the decomposition of the signal into time-blocks appears to be a key in the evaluation of the individual’s profile.

### Limitations

Considering the current available data, the proposed methodology does not explore the physiological reasons of the risk of fall. Due to the relative lack of realistic conditions in the quiet-stand acquisition protocol, previous works questioned the ability of such a measurement to provide physiological reasons of fall [38]. Considering that fragile individuals, depending on their impairement, have difficulty in processing simultaneous or specific tasks [6, 36], richer protocols have been proposed (including multi-tasking or use of foam surfaces) [3, 16, 38]. We agree with the arisen perspectives in terms of faller/non-faller classification as well as particular impairement’s investigation (visual, vestibular, somatosensor, nervous system). However, the objective of this work was to highlight the existence of UBs in both fallers’ and non-fallers’ statokinesigrams, and that UBs are more frequent in fallers than in non-fallers. We believe that richer protocols could improve further the classification and prediction performance of our approach.

A second limitation consists to the relative uncertainty about the physiological meaning of blocks characterized as “unquiet blocks” (UBs). There is no any guarantee that the blocks labeled statistically as possible UBs are indeed instabilities. Therefore, no direct links can be done between UBs and instant loss of balance control. Although global scores classify effectively fallers/non-fallers, clinical annotation and pre-fall recordings might be needed in order to understand and characterize better the UBs.

Furthermore, it is worth noting that during consultation, where participants were asked to declare falls in previous six months, some of them might confabulate without a conscious intention to deceive (recall bias). Therefore, minor part of the non-faller population might be mistakenly annotated. While the machine learning algorithms are robust to this problem, the latter annotation uncertainty might contribute to the prominent presence of UBs (faller-like profile) in some wrongly-annotated non-fallers.

Another limitation might be the choice of the Wii Balance Board as a force platform. Previous works have questioned the reliability of the Wii Balance Board in evaluation of posture [39]. Wii Balance Board was found to have a modest agreement with laboratory grade force platforms [40] as well as lower accuracy and lower signal to noise ratio compared to them. Moreover, irregular sampling rate is also listed in the drawbacks of using Wii Balance Board. Although we are aware about the latter disadvantages, Wii Balance Board presents an increasing popularity in posturography studies as a valid tool for assessing standing balance [23–25, 41]. In addition, recent works [24, 28] showed that careful pre-processing can even increase the accuracy of Wii Balance Board. We believe that more accurate force platforms, future expert’s annotation as well as richer protocols will provide a physiological multi-dimensional profile of UBs, reinforcing the physiological and methodological conclusions of the above approach. Further investigation should be done in methodologies which are able to fully exploit the richness of such protocols.

## Conclusions

In this study, a new approach for detecting parts of statokinesigram that are more close to unquiet blocks in an community-dwelling elderly population (126 individuals) was proposed. The results were confirmed using global scores from the above detection, which were able to classify fallers/non-fallers population. The approach separated statokinesigrams in time-blocks and provided a soft classification between two main clusters (UBs/QBs) based on the Expectation-Maximization algorithm(EM) for Gaussian Mixture Models (GMM). We showed that 1) separating the signals into predefined periods and applying a multi-dimensional analysis in smaller parts (time-blocks) of each statokinesigram rather than the whole signal, could highlight better the indicative periods of each statokinesigram and, 2) although unquiet blocks might exist in both faller and non-faller statokinesigrams, they are expected to be more frequent in fallers’ statokinesigrams rather than in those of non-fallers.

The proposed method can provide a supplementary indication about the risk of fall of an individual. Interested clinicians/researchers can find an online version of the resulting scoring algorithm(http://taureau.pppcmla.ens-cachan.fr/), which can be applied either to the existing dataset or to new statokinesigrams. We believe that the proposed method provides a promising methodology of dealing with complex signals like statokinesigrams. Other approaches in terms of indices’ selection or classification algorithms may be applied in order to increase the above proposed performance. The simplicity of acquisition protocol, the cost and portability (and the reasonable accuracy) of Wii Balance Board and the effectiveness of the proposed method, may be of major usefulness in increasing the investigated cohorts and expand the evaluation of unquiet periods in widespread-diseases such as Parkinson’s disease.

## Supporting information

S1 Appendix Comparison of algorithms when statokinesigrams are considered homogeneous. (PDF)

## Abbreviations

WBB: Wii Balance Board
QB: Quiet Block
UB: Unquiet Block
GMM: Gaussian Mixture Model
