# Supplementary material for: On the importance of local dynamics in statokinesigram: A multivariate approach for postural control evaluation in elderly
Source: PLoS One. 2018 Feb 23;13(2):e0192868. doi: 10.1371/journal.pone.0192868 (PMC5825048; doi:10.1371/journal.pone.0192868)
Supplement: S1 Appendix — (PDF) [file pone.0192868.s001.pdf]

S1 Appendix I

In order to highlight the importance of the unsupervised GMM approach (where no assumption is made on the nature of the block) compared to a regular supervised classification approach (where each block is supposed to possess the label of the whole signal), we performed additional experiments using support vector machine (SVM) classification with linear, polynomial and radial basis (RBF) kernel (choosing the same cross validation parameters as GMM). The performance of these classification models were $AUC_{SVM} \leq 0.70$ – a clear downgrade from the 0.77 of our simple GMM model. See details in Table S1 below:

Table S1: Classification performance of the global score using different block-lengths and two basic different classification strategies, unsupervised and supervised. The proposed Gaussian mixture model approach has clearly higher performance than every tested kernel function used by SVM.

Block-length	AUC (average ± std)	
	GMM (unsupervised)	Best kernel function SVM per block-length (supervised)
1 sec	0.77 ± 0.09	0.68 ± 0.12
2 sec	0.77 ± 0.10	0.70 ± 0.12
3 sec	0.76 ± 0.09	0.68 ± 0.12
Signal's duration	0.63 ± 0.12	0.69 ± 0.11
